# Supplementary material for: Global coincident bursts of high frequency oscillations across the human cortex coordinate large-scale memory processing
Source: Nat Commun. 2026 Mar 15;17:3996. doi: 10.1038/s41467-026-70633-7 (PMC13136343; doi:10.1038/s41467-026-70633-7)

Table S1. Recall performance and co-HFO bursting rate abundance across participants.
This table summarizes the total number of words recalled, recall rate (as a percentage of studied words), and rates of co-HFO bursts during the recall period for each participant. These values were used to assess the relationship between task performance and co-HFO activity, as reported in the main text.

**Subject**	**Recalled Words (n)**	**Recall Rate (%)**	**Rates of Co-HFO bursts (Hz / word)**
2	50	27.8	105.57
3	68	37.8	64.59
5	62	34.4	75.83
6	42	23.3	119.40
7	55	30.6	80.60
8	55	30.6	67.45
9	63	35	74.82
10	47	26.1	96.77
11	80	44.4	66.59
13	48	26.7	35.11
14	63	35	28.63
16	23	12.8	95.28
**Average**	54.3	30.7	48.55

Table S2. Distribution of electrode contacts implanted across cortical areas for treatment of drug-resistant epilepsy treatment.
Electrode placement varied across cortical regions based on clinical priorities, leading to non-uniform coverage across patients. The table summarizes the number of electrodes implanted per structure from all patients, reflecting variability in cortical sampling due to patient-specific clinical needs.

Structure	N electrodes
**Amygdala**	32
**Angular gyrus**	24
**Calcarine fissure**	4
**Caudate nucleus**	4
**Cingulate gyrus**	35
**Cuneus**	11
**Fusiform gyrus**	40
**Gyrus rectus**	17
**Heschl's gyrus**	10
**Hippocampus**	156
**Inferior frontal gyrus**	104
**Inferior occipital gyrus**	2
**Inferior parietal gyrus**	38
**Inferior temporal gyrus**	103
**Insula**	172
**Lingual gyrus**	31
**Middle occipital gyrus**	19
**Middle temporal gyrus**	313
**Orbitofrontal cortex**	56
**Paracentral lobule**	4
**Parahippocampal gyrus**	27
**Postcentral gyrus**	47
**Precentral gyrus**	38
**Precuneus**	37
**Putamen**	49
**Rolandic operculum**	42
**Superior frontal gyrus**	52
**Superior occipital gyrus**	10
**Superior parietal gyrus**	14
**Superior temporal gyrus**	124
**Supramarginal gyrus**	61
**Temporal Pole**	41

Figure S1. Detection and validation of high-frequency oscillations (HFOs).
a) Example raw signal with its decomposition into the studied ranges of high gamma (60–150 Hz), ripple (150–250 Hz), and fast ripple (250–600 Hz) frequencies. The amplitude envelope was extracted using the Hilbert transform and z-scored. Detected HFO bursts are highlighted by red boxes. The corresponding Hilbert matrix illustrates spectral power dynamics across the frequency scale and over time with the vertical white bars indicating the frequency span and the horizontal white bars the duration of each detection, respectively. b) Comparison of a synthetic broadband transient introduced into the signal, which was not detected by the algorithm, with a selectively detected oscillatory burst. Notice that the artifactual broadband event was not detected despite a relatively large amplitude and duration. c) Distribution of all HFO detections from an example recall trial shows a subpopulation of events with a higher frequency span (red points) relative to the majority of the detected events with a narrow frequency span (black points) identified by unsupervised clustering (k-means, k = 2, x mark the centroid of each cluster). Notice that excluding the subpopulation of higher frequency span did not affect the main results reported in the study. d) Comparison of the probability of co-HFO bursting (as in Fig. 2c) between the high-specificity subset of detections excluding events with larger frequency spans (left) and the full dataset (right). Shaded regions represent mean +/- SEM across N=12 biologically independent participants. Notice that excluding the subpopulation of higher frequency span events did not affect the main results reported in the study. e) Comparison of co-HFO bursting probabilities repeated using alternative correlation thresholds (r > 0.3 and r > 0.7) to test the robustness of the findings. Shaded regions represent mean +/- SEM across N=12 biologically independent participants. The temporal structure of co-HFO bursting across task phases remained stable across thresholds, consistent with the patterns described in the main text using the r > 0.5 threshold.

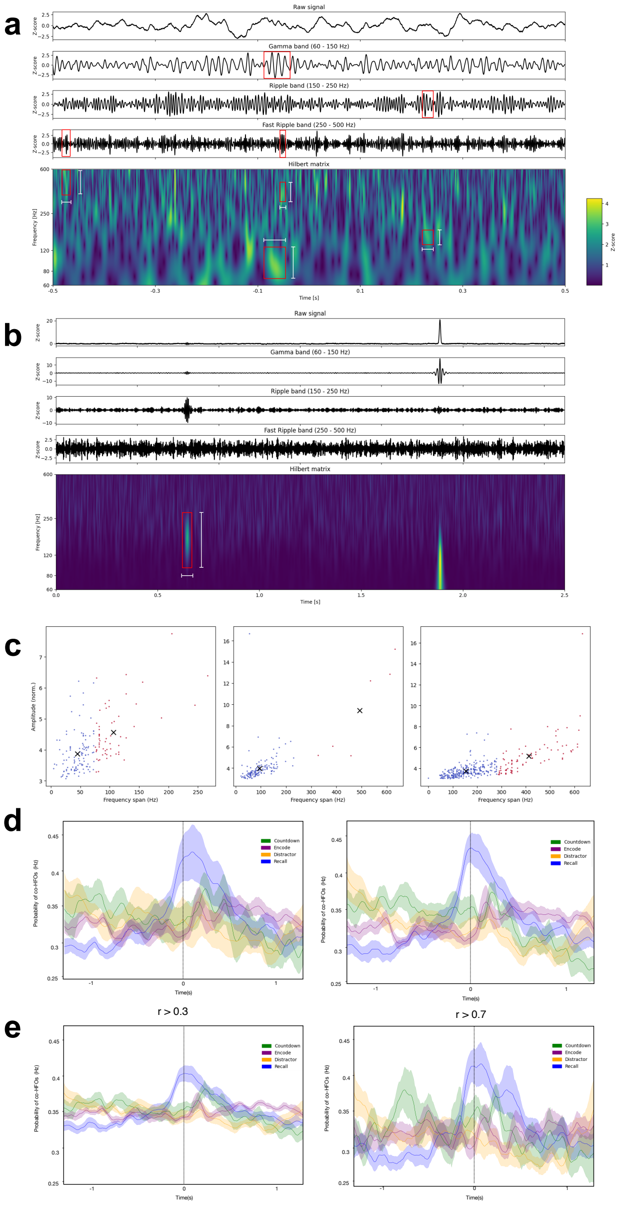

Figure S2 Dynamic Time Warping (DTW) alignment of verbal responses with audio processing. (A) Example of one DTW-based alignment of spoken words with their corresponding timestamps (red lines) is presented on a spectrogram of the recorded patient audio responses. The bottom panel presents the Mel-Frequency Cepstral Coefficients (MFCC) spectrogram of the audio signal. (B) Verbal responses were first automatically annotated using a fine-tuned Whisper model and then manually supervised and corrected, if needed, with timestamp realignment to ensure precise onset of the very beginning of word vocalization.

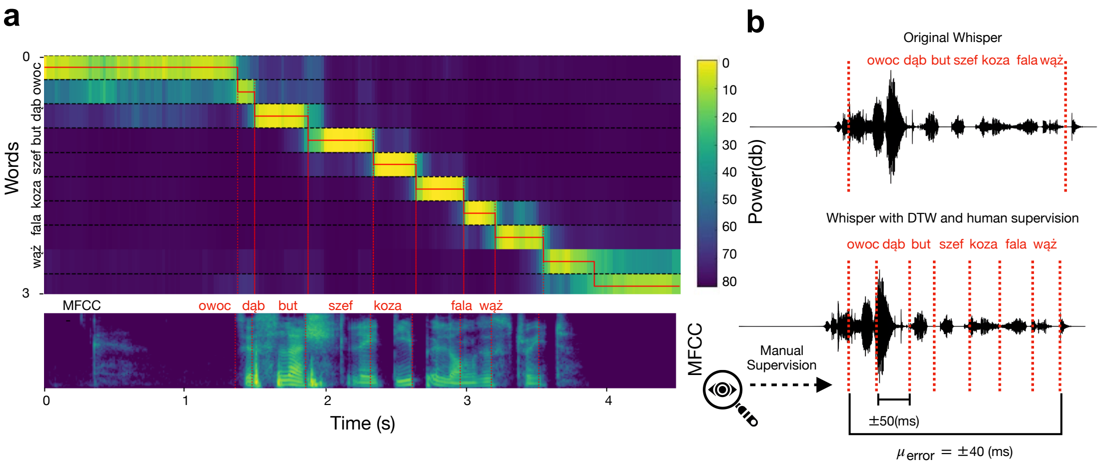

Figure S3. Baseline HFO bursting rates show no differences across task phases. Bursting rates per frequency range (60–150 Hz, 150–250 Hz, and 250–500 Hz) across the color-coded task phases reveal no significant differences (Kruskal-Wallis test, H(3) = 2.94, p = 0.40, N = 12 biologically independent participants) and consistent rates across patients (points) in all three frequency ranges. Error bars indicate standard deviation.

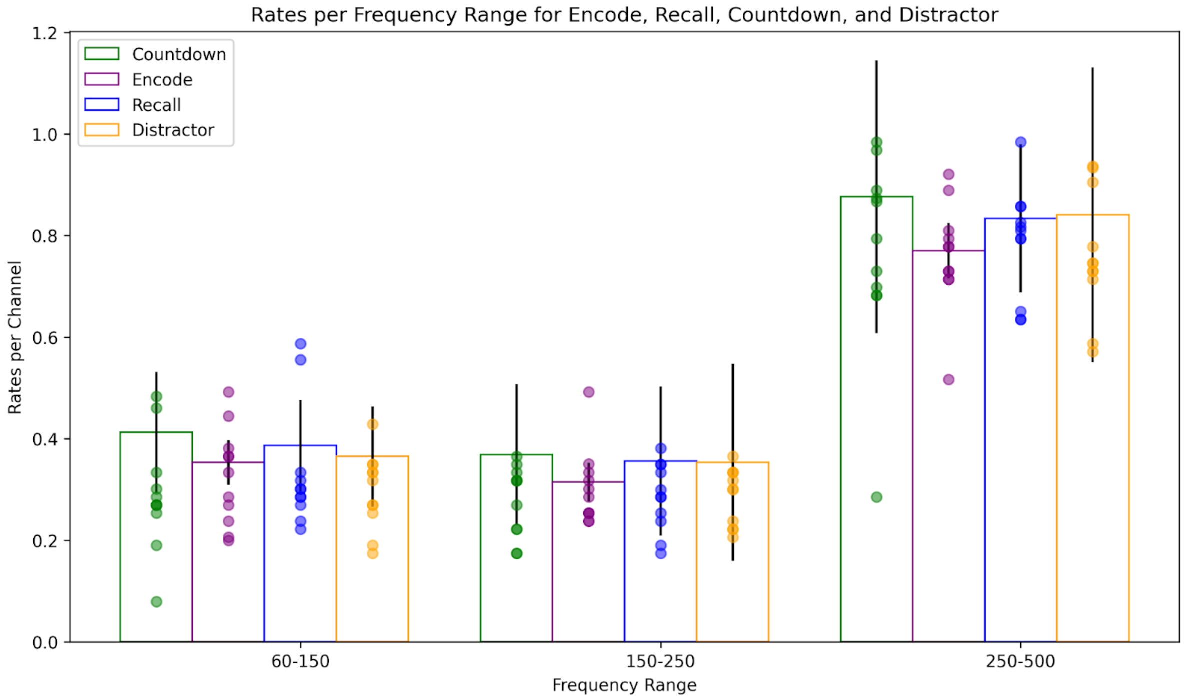

Fig. S4.Pairwise condition contrasts of co-HFO bursting dynamics across time. Using linear mixed-effects modeling, all six comparisons between task conditions (Recall, Countdown, Encode, Distractor) were performed by rotating the reference level for Condition while controlling for subject as a random effect. FDR-corrected q-values (two-sided) are plotted across time bins for each condition pair (analysis derived from N = 12 biologically independent participants).

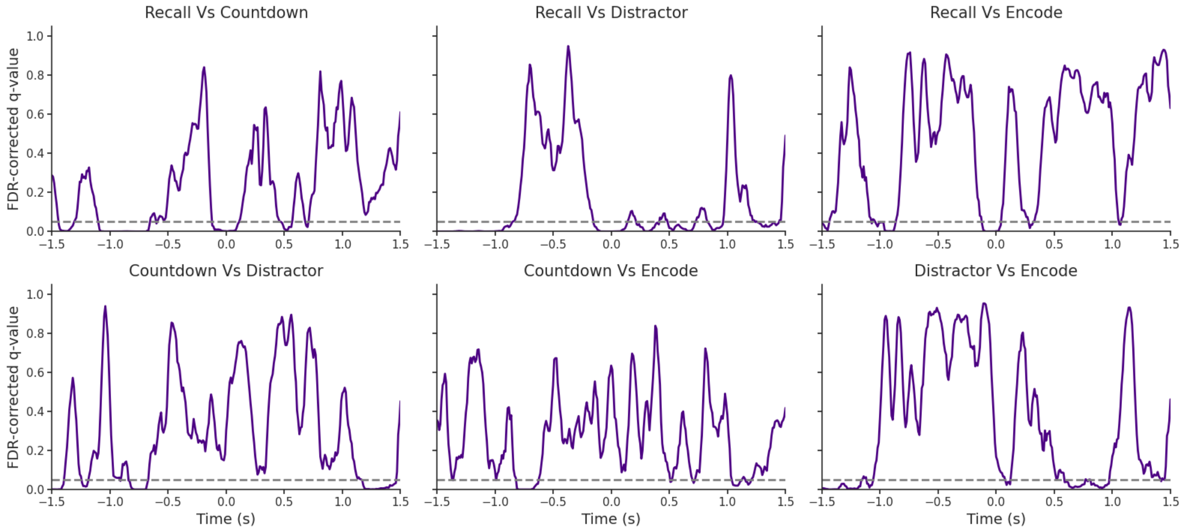

Figure S5. Co-occurrence of HFO bursts across cortical regions shows consistent differences between trials with subsequently remembered and forgotten words. Probabilities of co-HFO bursting, plotted as in Fig. 3b, for each lobe (row) and hemisphere (column) revealed the greatest differences (black arrows) in the right limbic and frontal lobes. Notice that in all cases the probability sharply decreases just before word presentation (time 0), followed by increased bursting during the time of word processing from around 150–1000 ms. Shaded regions represent mean +/- SEM across N = 12 biologically independent participants.

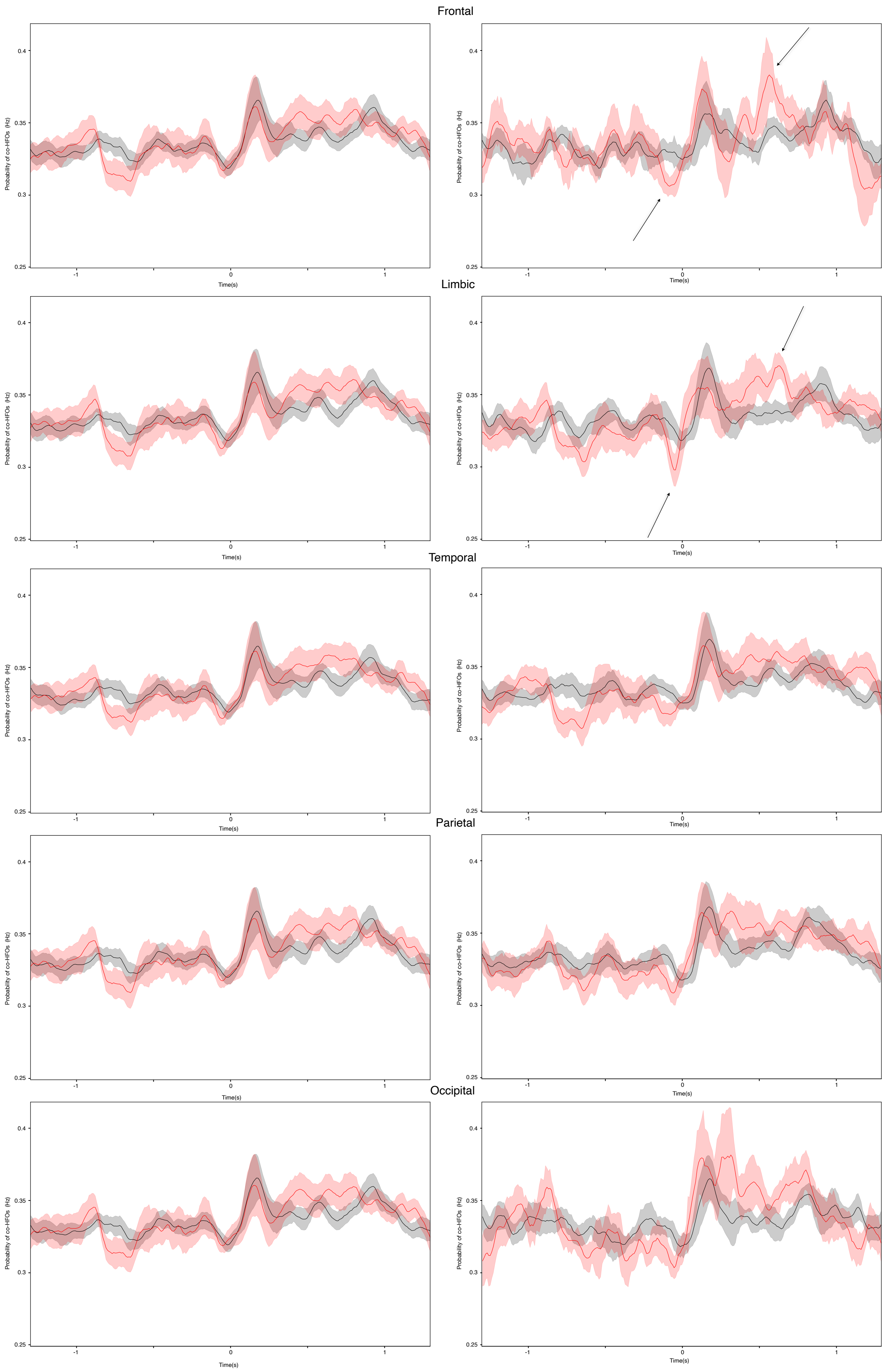

Figure S6. Summary of the channel overlap during encoding and recall of the same word. Bar plots show the proportion of channels exhibiting co-HFO bursting during encoding and recall of each word (x-axis) across the 12 biologically independent participants(each panel is one patient). Notice that despite wide variability in the proportion of overlapping channels and the words recalled, more than 10% of all contacts were on average engaged in co-HFO bursting during encoding and recall of the same word.

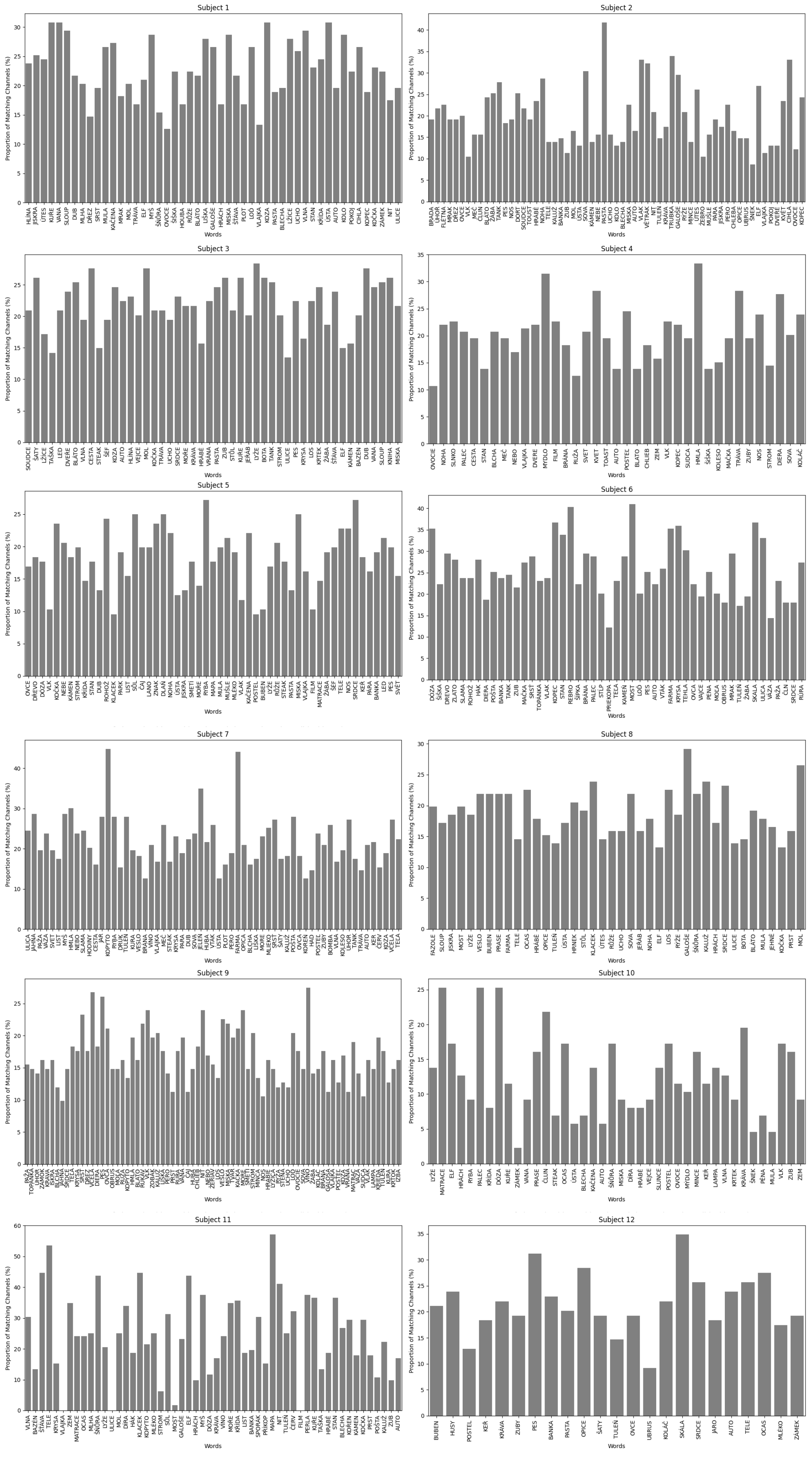

Fig. S7. Surrogate jitter analysis reveals temporally structured co-HFO bursting during encoding and recall. a) Word-level differences in mean synchrony (original minus jittered) across recalled words for a representative subject (subject 2). b) Distributions of mean pairwise synchrony values (Pearson correlation across co-HFO matrices) for original and jittered datasets during encoding and recall.

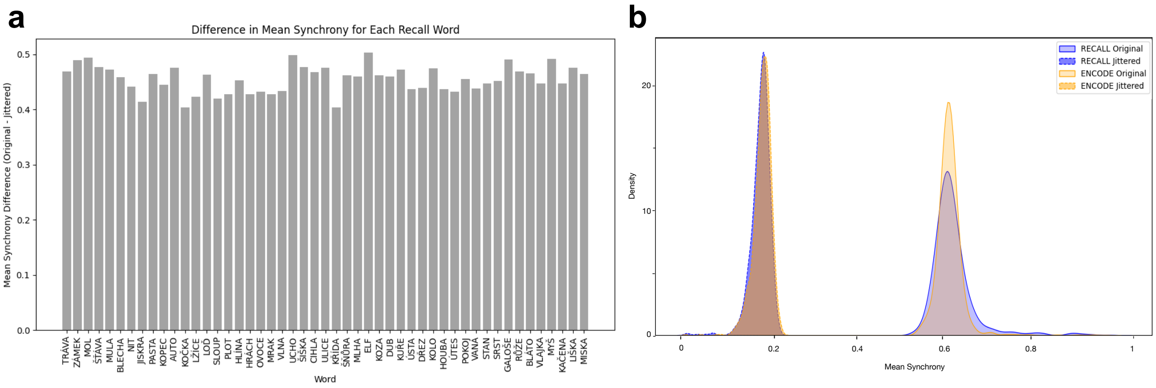

Figure S8. Per-subject visualization of the percent involvement of each implanted electrode contact in co-HFO bursting during word recall. Each dot represents an electrode contact, color-coded by the proportion of recalled words in which it participated in a co-HFO burst.Data is shown for N = 12 biologically independent participants.

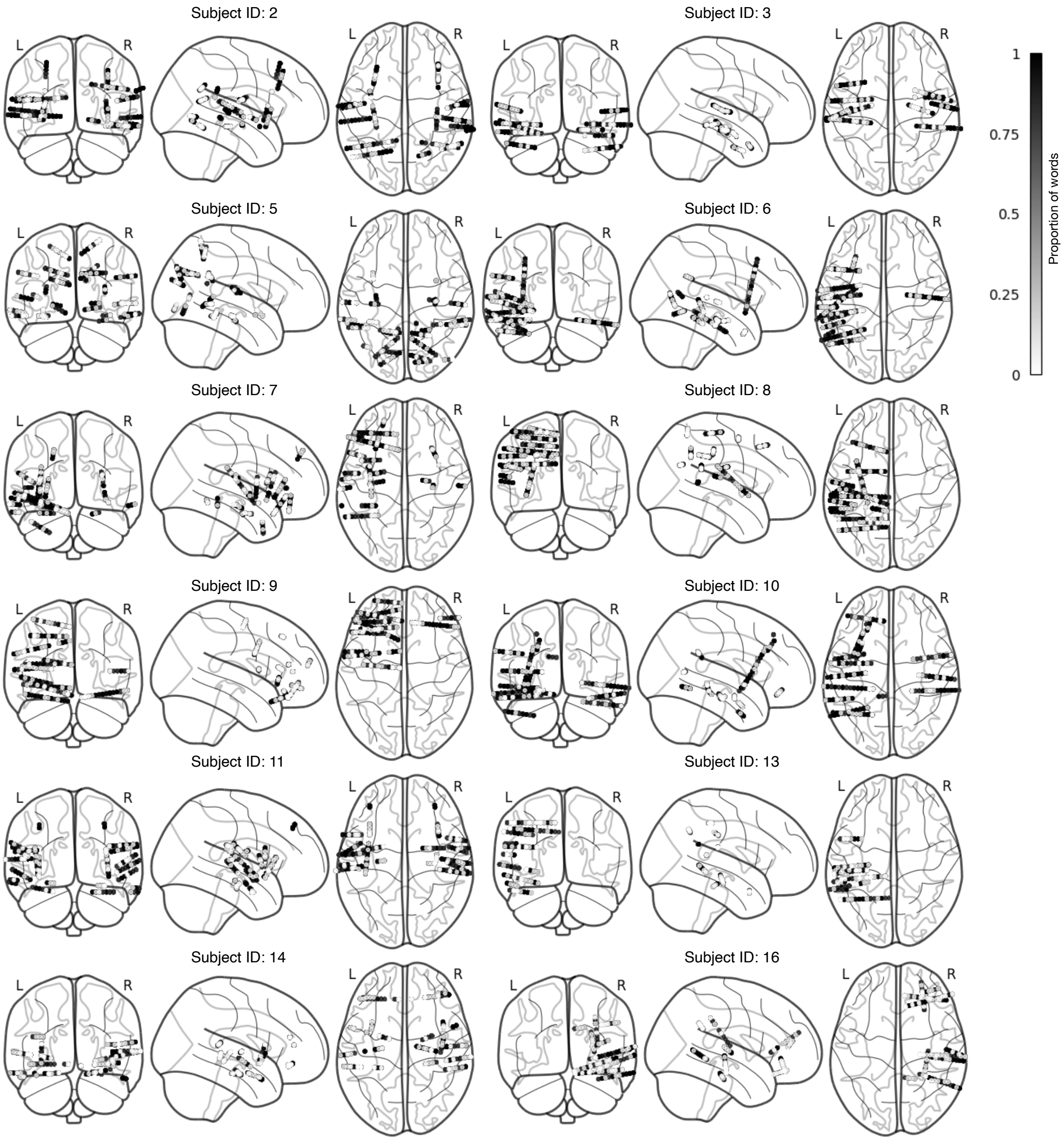

Fig S9. Comparison of the global co-HFO bursting dynamics between electrode contacts localized in the gray and white matter. a) Mean co-HFO bursting across the significantly correlated channel pairs plotted as in Fig. 2c but restricted to gray matter contacts shows consistent increases around recall onset and during word presentation (N = 12 biologically independent participants). b) The same analysis using only white matter contacts reveals analogous increases but less pronounced and more variable (N = 8 biologically independent participants) (black bars indicate significant bins identified by the linear mixed-effects model (LMM), two-sided P < 0.05, FDR-corrected). Shaded regions represent mean +/- SEM.

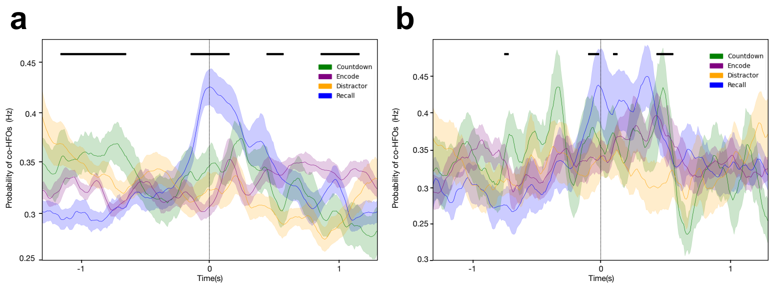

Supplement: Supplementary file 1 — Supplementary Information [file 41467_2026_70633_MOESM1_ESM.pdf]
