# Supplementary material for: Global coincident bursts of high frequency oscillations across the human cortex coordinate large-scale memory processing
Source: Nat Commun. 2026 Mar 15;17:3996. doi: 10.1038/s41467-026-70633-7 (PMC13136343; doi:10.1038/s41467-026-70633-7)
Supplement: Supplementary file 2 — Reporting Summary [file 41467_2026_70633_MOESM2_ESM.pdf]

Corresponding author(s): Michal Kucewicz

Last updated by author(s): Jan 30, 2026

Reporting Summary

Nature Portfolio wishes to improve the reproducibility of the work that we publish. This form provides structure for consistency and transparency in reporting. For further information on Nature Portfolio policies, see our Editorial Policies and the Editorial Policy Checklist.

Statistics

For all statistical analyses, confirm that the following items are present in the figure legend, table legend, main text, or Methods section.

n/a	Confirmed	
☐	☒	The exact sample size (*n*) for each experimental group/condition, given as a discrete number and unit of measurement
☐	☒	A statement on whether measurements were taken from distinct samples or whether the same sample was measured repeatedly
☐	☒	The statistical test(s) used AND whether they are one- or two-sided<br>*Only common tests should be described solely by name; describe more complex techniques in the Methods section.*
☒	☐	A description of all covariates tested
☐	☒	A description of any assumptions or corrections, such as tests of normality and adjustment for multiple comparisons
☐	☒	A full description of the statistical parameters including central tendency (e.g. means) or other basic estimates (e.g. regression coefficient) AND variation (e.g. standard deviation) or associated estimates of uncertainty (e.g. confidence intervals)
☐	☒	For null hypothesis testing, the test statistic (e.g. *F*, *t*, *r*) with confidence intervals, effect sizes, degrees of freedom and *P* value noted<br>*Give P values as exact values whenever suitable.*
☒	☐	For Bayesian analysis, information on the choice of priors and Markov chain Monte Carlo settings
☒	☐	For hierarchical and complex designs, identification of the appropriate level for tests and full reporting of outcomes
☐	☒	Estimates of effect sizes (e.g. Cohen's *d*, Pearson's *r*), indicating how they were calculated

Our web collection on statistics for biologists contains articles on many of the points above.

Software and code

Policy information about availability of computer code

Data collection

Electrophysiological Recordings:

Data were collected using two commercial intracranial EEG (iEEG) acquisition systems:
• ATLAS neurophysiological recording system (FHC-Neuralynx Inc., 32,000 Hz sampling rate)
Used at Jan Mikulicz-Radecki University Hospital in Wroclaw.
• BrainScope BioSDA09 (M&I Ltd., 5,000 Hz sampling rate)
Used at St. Anne's University Hospital in Brno.

These systems recorded signals from up to 128 or 192 channels, respectively, using standard depth electrodes (Ad-Tech Inc.) as part of clinical stereo-EEG implantation for seizure monitoring.

Task Presentation:

Behavioral memory tasks (Free Recall and Paired Associate Learning) were programmed and presented using a custom-developed interface and TTL pulse output from the task computer was used to synchronize task events with neural recordings.

Audio and Speech Data:
• Microphone Hardware: High-fidelity digital microphones were used to capture verbal responses during memory recall tasks.
• Speech-to-Text Transcription: Responses were transcribed using a custom transcription interface based on a fine-tuned version of the

Whisper ASR model (OpenAI Whisper v1.1) with Dynamic Time Warping for alignment.

Imaging:
• CT and MRI Scans were acquired using hospital-standard clinical imaging protocols.
• Electrode localization was performed by co-registering pre-operative CT and post-operative MRI scans using standard neuroimaging toolkits, followed by normalization to MNI space and anatomical labeling with the AAL3 atlas.

Data analysis

Custom-made software:
A custom Python-based analysis pipeline was developed specifically for this study to process, analyze, and visualize High-Frequency Oscillations (HFOs) in intracranial EEG data. This pipeline, named coincident hfo is available at: https://gitlab.com/brainandmindlab/coincident-hfo.git and requires Python 3.8+.
It includes scripts for data organization, segmentation into trial-specific HFO events, statistical comparison of HFO synchronization, and brain map visualizations.
• HFO Detection Software:
HFO events were detected using the open-source library epycom (https://github.com/ICRC-BME/epycom), which provides signal processing routines for electrophysiological data.
• Python Libraries (Open Source):
• numpy (v1.24 or later)
• pandas (v1.5 or later)
• matplotlib (v3.6 or later)
• seaborn (v0.12 or later)
• nilearn (v0.10 or later) – for neuroimaging visualization
• statsmodels (v0.13 or later) – for statistical tests
• argparse, pickle – standard Python modules used for argument parsing and data serialization.
• Electrophysiological data format and structure:
Data were stored in Multiscale Electrophysiology Format (MEF) v3.6 and organized according to the BIDS (Brain Imaging Data Structure) standard to ensure compatibility and reproducibility.

For manuscripts utilizing custom algorithms or software that are central to the research but not yet described in published literature, software must be made available to editors and reviewers. We strongly encourage code deposition in a community repository (e.g. GitHub). See the Nature Portfolio guidelines for submitting code & software for further information.

Data

Policy information about availability of data

All manuscripts must include a data availability statement. This statement should provide the following information, where applicable:

- Accession codes, unique identifiers, or web links for publicly available datasets
- A description of any restrictions on data availability
- For clinical datasets or third party data, please ensure that the statement adheres to our policy

The Human brain local field potential recordings during a battery of multilingual cognitive and eye-tracking tasks (v1) data used in this study are available in the EBRAINS database at https://doi.org/10.25493/4FZH-ZCG. All analyses were conducted on data from this openly accessible resource. The raw LFP data are protected and are not available in the public repository due to data privacy laws regarding human participants. The processed data used in this study are available at EBRAINS. Source data for all plots and figures generated in this study are provided as a Source Data file with this paper.

Research involving human participants, their data, or biological material

Policy information about studies with human participants or human data. See also policy information about sex, gender (identity/presentation), and sexual orientation and race, ethnicity and racism.

Reporting on sex and gender

Sex of participants was determined based on self-reporting during clinical intake, in accordance with hospital policy. Gender identity was not recorded, and no gender-specific analyses were conducted. The final cohort included 17 patients, of whom 10 identified as male and 7 as female (see Table 1). All were undergoing intracranial EEG monitoring for epilepsy surgery, and provided written informed consent for participation and data sharing. The mean age of participants was 31.94 ± 1.63 years. Sex-based demographic data (sex, age) and trial counts are reported in the source data. However, no sex-based or gender-based analyses were performed, as the primary goal of the study was to evaluate high-frequency oscillatory synchronization patterns related to cognitive task structure, not individual differences. Given the small and unbalanced sample size, statistical power was insufficient to support meaningful sex-based subgroup comparisons. Therefore, sex was not included as a variable in the main analyses.
Consent for sharing of individual-level data was obtained, and participant data have been anonymized. Individual subject-level sex and age data are available in the source data.

Reporting on race, ethnicity, or other socially relevant groupings

In this study, the only socially relevant categorization variable used was sex, as self-reported by participants during clinical intake procedures. Gender identity, ethnicity, race, and socioeconomic status were not collected, and thus were not analyzed or used as proxies for any other variable.
Participants were categorized into male and female based on self-identification, following hospital admission protocols. No external or administrative data sources were used for categorization. These labels were used solely for descriptive reporting of cohort demographics (e.g., Table 1) and were not included as factors in statistical analysis, due to limited sample size and unequal group representation (10 male, 7 female). The study does not make claims regarding differences between social

groups, and no assumptions were made regarding other social categories or identities.

Population characteristics	Seventeen human participants (10 male, 7 female; mean age = 31.94 ± 1.63 years) undergoing intracranial stereo EEG monitoring for treatment-resistant epilepsy were included in this study. All participants were surgical candidates recruited from St. Anne's University Hospital in Brno and Jan Mikulicz-Radecki University Hospital in Wroclaw. Diagnosis of drug-resistant epilepsy was confirmed in all cases. Electrode implantation and task participation were conducted during their clinical monitoring period. Participants were native speakers of Czech (n=8), Slovak (n=4), or Polish (n=5). No genotypic information was collected. Participants were not selected based on specific etiology, seizure type, or prior treatment history. Electrode coverage varied based on individual clinical needs.
Recruitment	Participants were recruited from the clinical population of epilepsy patients undergoing intracranial stereo EEG monitoring as part of their standard pre-surgical evaluation at St. Anne's University Hospital in Brno and Jan Mikulicz-Radecki University Hospital in Wroclaw. Inclusion was based on clinical need for electrode implantation, not research-specific criteria. Participation in the cognitive tasks was voluntary, and written informed consent was obtained from all individuals. Because recruitment was limited to surgical candidates with drug-resistant epilepsy, there is a potential selection bias toward patients with more severe or treatment-resistant forms of epilepsy.
Ethics oversight	The study protocol, including participant recruitment, consent procedures, and data handling, was approved by the Institutional Review Boards and Ethics Committees of:<br>• St. Anne's University Hospital, Brno, Czech Republic, and<br>• Jan Mikulicz-Radecki University Hospital, Wroclaw, Poland.<br>Ethical approval details are provided in the manuscript, and all procedures adhered to the Declaration of Helsinki and local regulatory requirements for human research.

Note that full information on the approval of the study protocol must also be provided in the manuscript.

Field-specific reporting

Please select the one below that is the best fit for your research. If you are not sure, read the appropriate sections before making your selection.

☒ Life sciences ☐ Behavioural & social sciences ☐ Ecological, evolutionary & environmental sciences

For a reference copy of the document with all sections, see nature.com/documents/nr-reporting-summary-flat.pdf

Life sciences study design

All studies must disclose on these points even when the disclosure is negative.

Sample size	No formal statistical power analysis was conducted to predetermine sample size. The sample size of 17 participants (after excluding 5 for technical issues) was determined based on the availability of patients undergoing stereo EEG monitoring at the two participating hospitals during the recruitment period. This sample size is in line with previous comparable studies in human intracranial electrophysiology, where clinical constraints limit larger cohort sizes. The number of trials per participant and per task condition provided sufficient within-subject statistical power for time-resolved and correlation-based analyses.
Data exclusions	Data from five participants were excluded from all analyses due to technical issues that caused unreliable synchronization between task event timing and electrophysiological recordings. These exclusion criteria were pre-established, as precise event alignment was essential for the intended analyses of HFO synchronization. No other data exclusions were made.
Replication	The analyses included multiple task runs (Free Recall and Paired Associate Learning) across participants, with repeated trials in each condition. Findings were assessed for consistency within and across participants and validated using established statistical methods . While independent replication in a separate dataset is beyond the scope of this clinical study, results were internally reproducible across trials and tasks.
Randomization	Randomization was not applicable to the allocation of participants, as electrode implantation and recording site decisions were made based on clinical needs. However, stimuli (word lists and word pairs) were randomized in their presentation order within and across trials to avoid order effects and memory priming
Blinding	Blinding was not feasible due to the nature of the study. Participants were aware of the task being performed, and researchers conducting the electrophysiological data analysis had access to trial and condition labels. However, quantitative analyses were based on objective signal features and automated processing pipelines, minimizing bias.

Reporting for specific materials, systems and methods

We require information from authors about some types of materials, experimental systems and methods used in many studies. Here, indicate whether each material, system or method listed is relevant to your study. If you are not sure if a list item applies to your research, read the appropriate section before selecting a response.

Materials & experimental systems

n/a	Involved in the study
☒	☐ Antibodies
☒	☐ Eukaryotic cell lines
☒	☐ Palaeontology and archaeology
☒	☐ Animals and other organisms
☒	☐ Clinical data
☒	☐ Dual use research of concern
☒	☐ Plants

Methods

n/a	Involved in the study
☒	☐ ChIP-seq
☒	☐ Flow cytometry
☒	☐ MRI-based neuroimaging

Plants

Seed stocks

Report on the source of all seed stocks or other plant material used. If applicable, state the seed stock centre and catalogue number. If plant specimens were collected from the field, describe the collection location, date and sampling procedures.

Novel plant genotypes

Describe the methods by which all novel plant genotypes were produced. This includes those generated by transgenic approaches, gene editing, chemical/radiation-based mutagenesis and hybridization. For transgenic lines, describe the transformation method, the number of independent lines analyzed and the generation upon which experiments were performed. For gene-edited lines, describe the editor used, the endogenous sequence targeted for editing, the targeting guide RNA sequence (if applicable) and how the editor was applied.

Authentication

Describe any authentication procedures for each seed stock used or novel genotype generated. Describe any experiments used to assess the effect of a mutation and, where applicable, how potential secondary effects (e.g. second site T-DNA insertions, mosiacism, off-target gene editing) were examined.
